# Planning, performing and analyzing X-ray Raman scattering experiments

**DOI:** 10.1107/S1600577514027581

**Published:** 2015-02-03

**Authors:** Ch. J. Sahle, A. Mirone, J. Niskanen, J. Inkinen, M. Krisch, S. Huotari

**Affiliations:** a Department of Physics, PO Box 64, FI-00014 University of Helsinki, Helsinki, Finland; b European Synchrotron Radiation Facility, BP 220, F-38043 Grenoble Cedex, France

**Keywords:** X-ray Raman scattering, inelastic X-ray scattering, spectroscopy, direct tomography

## Abstract

A compilation of procedures for planning and performing X-ray Raman scattering (XRS) experiments and analyzing data obtained from them is presented. In particular, it is demonstrated how to predict the overall shape of the spectra, estimate detection limits for dilute samples, and how to normalize the recorded spectra to absolute units. In addition, methods for processing data from multiple-crystal XRS spectrometers with imaging capability are presented, including a super-resolution method that can be used for direct tomography using XRS spectra as the contrast. An open-source software package with these procedures implemented is also made available.

## Abstract 2

A summarising review of data treatment for non-resonant inelastic X-ray scattering data from modern synchrotron-based multi-analyzer spectrometers.

## 1. Introduction   

X-ray Raman scattering (XRS) is non-resonant inelastic X-ray scattering (IXS) from core-electron excitations. Over the past decades XRS spectroscopy has become a valuable tool to study electronic excitations in crystalline (Mattila *et al.*, 2005 ▸; Sternemann *et al.*, 2008*a* ▸; Pylkkänen *et al.*, 2010 ▸; Huotari *et al.*, 2012 ▸; Nyrow *et al.*, 2014*a* ▸,*b* ▸; Tse *et al.*, 2014 ▸; Pascal *et al.*, 2014 ▸) and weakly ordered solids (Conrad *et al.*, 2009 ▸; Galambosi *et al.*, 2006 ▸) as well as liquids (Pylkkänen *et al.*, 2010 ▸, 2011 ▸; Wernet *et al.*, 2004 ▸; Sahle *et al.*, 2013 ▸; Juurinen *et al.*, 2013 ▸, 2014 ▸) and gases (Inkinen *et al.*, 2013 ▸; Sakko *et al.*, 2011 ▸).

The advent of third-generation synchrotron radiation facilities and especially the recent development of new XRS instruments at various light sources (Cai *et al.*, 2004 ▸; Fister *et al.*, 2006 ▸; Verbeni *et al.*, 2009 ▸; Sokaras *et al.*, 2012 ▸) has made this technique available to a broad range of users who apply XRS in a wide range of fields, from fundamental physics (Abbamonte *et al.*, 2004 ▸; Sternemann *et al.*, 2005 ▸; Weissker *et al.*, 2006 ▸) to materials sciences (Feroughi *et al.*, 2010 ▸) and geosciences (Lee *et al.*, 2005 ▸, 2008 ▸; Sternemann *et al.*, 2013 ▸). What makes XRS unique and so valuable is the ability to obtain soft X-ray absorption spectra (XAS), *i.e.* core-electron excitation spectra with energies 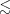
1 keV, while using hard X-rays in the experiment. Such XAS spectra include the *K*-edges of second-row elements, *L*- and *M*-edges of 3*d* transition metals, all the way to *O*-edges of the rare-earth elements and actinides. Thus, it provides a truly bulk sensitive probe to study these absorption edges, even for samples that are contained in complicated sample environments, such as catalytic reactors and diamond anvil cells (Mao *et al.*, 2003 ▸; Shieh *et al.*, 2013 ▸; Ding *et al.*, 2014 ▸).

A whole new usage of non-resonant inelastic X-ray scattering is emerging in the field of warm dense matter research, where non-resonant IXS (or X-ray Thomson scattering as it is called by this community) is used as an experimental diagnostics tool (Kritcher *et al.*, 2008 ▸; Glenzer & Redmer, 2009 ▸) to infer density and temperature of the samples at study. New large-scale facilities are currently under construction such as the large-scale laser facilities LLE and NIF or the Matter in Extreme Conditions (MEC) endstation at LCLS. As was shown by Mattern & Seidler (2013 ▸), careful analysis of the experimental data from these types of experiments is needed for an accurate description of the according equation of state.

However, the non-resonant IXS cross section is orders of magnitude smaller than that of photoelectric absorption. The low cross section demands high-brilliance beamlines with efficient spectrometers that cover a large solid angle of detection. XRS spectrometers are often based on spherically curved analyzer crystals that act as focusing monochromators after the sample and offer an energy resolution in the sub-eV range for photon energies ∼10 keV. Several modern IXS beamlines (Cai *et al.*, 2004 ▸; Fister *et al.*, 2006 ▸; Verbeni *et al.*, 2009 ▸; Sokaras *et al.*, 2012 ▸) provide large-solid-angle XRS spectrometers by utilizing dozens of such analyzer crystals. The XRS spectra depend on the momentum transferred to the system, and thus on the scattering angle realised in the experiment. Therefore, the scattering signal from each of the many analyzer crystals needs to be considered individually. Given that state-of-the-art beamlines have ever-increasing numbers of analyzer crystals installed (Cai *et al.*, 2004 ▸; Fister *et al.*, 2006 ▸; Verbeni *et al.*, 2009 ▸; Sokaras *et al.*, 2012 ▸), this poses a challenge for the experimentalist during the data analysis.

In this article, we describe ways to plan and perform XRS experiments, and analyze data obtained from them using the new multi-analyzer XRS spectrometers. We propose a methodology to evaluate the feasibility of an XRS experiment easily and systematically. We describe how using two-dimensional detectors simplifies XRS experiments, and how to extract the desired core excitation spectra from the measured total spectra.

The article is organized as follows: first, we will give a short overview of the theoretical background in §2, after which we describe how to plan experiments and evaluate their feasibility in §3. In §4 we outline the basic data processing that needs to be performed, and in §5 we describe the extraction of the desired shallow core edge spectra from the measured raw data. In §6 the direct tomography method is described and an approach to increase the spatial resolution of direct tomography images is presented. The article concludes with a summary and an outlook. Throughout this article, we use atomic units with 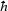 = 1; however, energies are expressed in electron volts (eV) and momenta in inverse ångstroms (Å^−1^) if convenient. Our examples are taken using the XRS spectrometers at the beamlines ID16^1^ and ID20 of the European Synchrotron Radiation Facility (ESRF; Grenoble, France), but the analysis is applicable to data taken using any other analyzer-crystal spectrometer as well.

## 2. Theoretical background   

**Figure 1:**
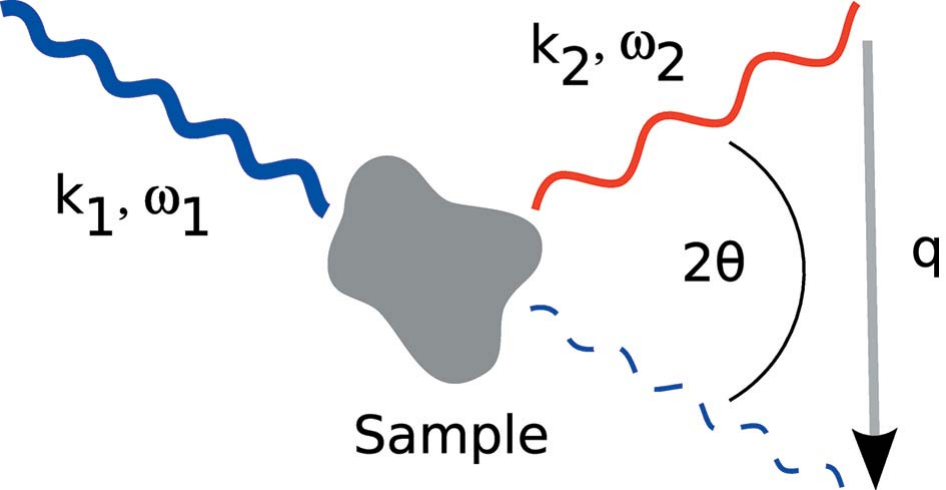
Schematic drawing of the principle of an IXS experiment. See text for details.

Fig. 1 ▸ shows the principle of an IXS experiment. A photon with energy 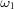 interacts with an electron of the sample and is scattered inelastically, emerging with a reduced energy 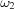. In the impact, the energy 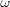 = 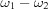 and the momentum 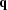 = 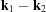 are transferred to the sample. Here 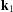 and 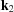 are the momenta of the incident and scattered photons, respectively. The absolute value of the momentum transfer 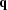 in equation (1) is given by 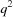 = 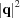 = 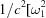 + 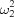 − 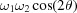], with the scattering angle 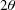. In XRS, the energy loss ω is tuned in the vicinity of soft X-ray absorption edges by varying 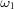, while observing the intensity of photons that emerge from the sample with a fixed 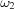 into the finite solid angle element dΩ. Most commonly, both incoming and scattered photon energies are of the order of 10 keV (*i.e.* hard X-rays).

In non-resonant IXS experiments, the incident X-ray photon energy is far away from any electron binding energy of the system and thus only the 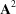 term of the electron–photon-interaction Hamiltonian contributes. The measured quantity is described by the double differential scattering cross section (DDSCS), which can be written as (Schülke, 2007 ▸)
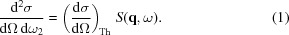
Here, 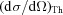 is the Thomson scattering cross section. In equation (1), 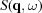 is the so-called dynamic structure factor and contains all information about the scattering many-body system that can be obtained by non-resonant IXS.

Excitations of a many-particle ground state 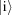 into all final states 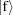 can be expressed *via* Fermi’s golden rule as
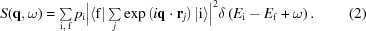
Here, 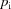 is the probability of finding the system in the initial state 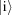 and the delta function ensures energy conservation.

Throughout this article, we will refer to background owing to the scattering from valence electrons. In order to appreciate this part of IXS, we note that 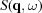 can be written as
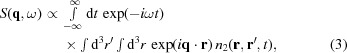
where 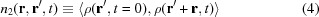
and 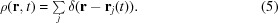
Here, 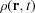 is the density operator for all electrons *j* at 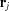 and time *t*. Thus, IXS allows direct access to the time-dependent two-particle density correlation function 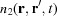. Equation (3) is an expression of the fluctuation-dissipation theorem, connecting inelastic scattering from time-dependent electron density fluctuations with excitations of the system (Schülke, 2007 ▸).

Representations (3) and (2) of 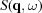 are equivalent but both stress different aspects giving access to the different branches or flavors of non-resonant IXS. In the case 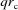 < 1 and 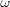 ≃ 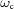 (where 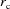 is some characteristic interparticle distance, and 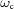 is, for example, the plasmon frequency), scattering by valence electron density fluctuations is probed and (3) becomes an intuitive representation of the measured quantity. If ω is tuned to match a core-electron excitation energy, scattering by that particular core electron is probed. This is the XRS branch of IXS and Fermi’s golden rule (2) becomes an intuitive representation.

The transition operator in (2) can be expanded as a Taylor series for small 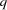 as 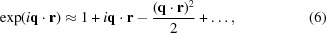 or, equivalently, in terms of spherical harmonics 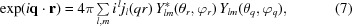
where 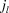 are spherical Bessel functions, 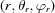 and 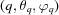 are the spherical coordinates of the vectors 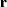 and 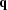, respectively, and 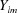 are spherical harmonics.

From equation (6), it is evident that at low *q* the XRS cross section is dominated by dipole transitions (second term) and then XRS becomes equivalent to X-ray absorption spectroscopy (XAS). However, in XRS the direction of 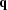 takes the role of the photon polarization vector in XAS. As *q* is increased, higher terms in the Taylor series (6) gain significance and XRS becomes sensitive to dipole forbidden transitions, such as the high-order multiplet features in the 3*d* transition metals, rare earths and actinides (Gordon *et al.*, 2008 ▸; Gupta *et al.*, 2011 ▸). Owing to this, the entire unoccupied density of states is accessible with XRS (Soininen *et al.*, 2005 ▸; Inkinen *et al.*, 2014 ▸). The nomenclature for these transitions (‘dipole’, ‘quadrupole’, *etc*.) follows from equation (7).

For very large transferred momenta *q*, *i.e.* if 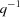 is small compared with characteristic interparticle distances, the scattering takes place within the so-called impulse approximation (IA) (Eisenberger & Platzman, 1970 ▸; Cooper *et al.*, 2004 ▸), and single particle properties are probed. This branch of IXS is called Compton scattering. In this case the measured spectrum is related to the Compton profile 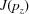, 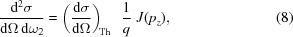
which, in turn, is the one-dimensional projection of the electron momentum density (Cooper *et al.*, 2004 ▸),
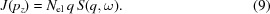
Here, 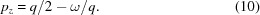
is the component of the scattered electron’s ground-state momentum 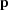 in the direction of the momentum transfer 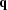 (conventionally taken to be along the 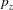-axis). In equation (9), 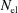 is the number of electrons in the atomic shell in question. The analysis of Compton scattering data has been reviewed by Huotari *et al.* (2009 ▸).

Within the scope of this article, the desired quantities are the spectra of inner-shell electronic excitations, *i.e.* XRS spectra. The XRS spectral onset (colloquially, and from hereon in this article, called ‘edge jump’) can be viewed as the onset of the Compton profile of the corresponding core electron. In particular, if *q* is large enough and at ω far above the XRS threshold, the IA begins to be valid and the high-energy tail of XRS spectra is equal to the Compton profile of the corresponding electron shell. This highlights the equivalence of XRS and Compton spectroscopy.

**Figure 2:**
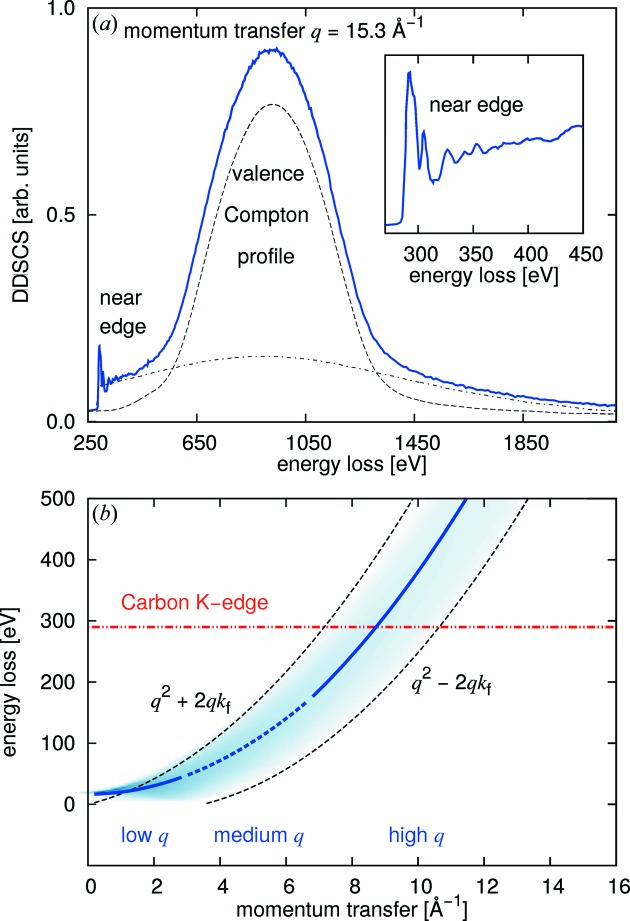
(*a*) Measured spectrum from polycrystalline diamond with 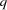 = 15.3 Å^−1^ [data taken from Huotari *et al.* (2012 ▸)]. The response of the valence electrons is clearly visible as a broad peak centered at 950 eV. The inset depicts a zoom into the carbon *K*-edge region. (*b*) Dispersion of the valence electron response (blue curves). To clarify what we mean by ‘low’, ‘medium’ and ‘high’ momentum transfer, the line is divided into three regions (solid blue for low and high momentum transfer, dashed for medium momentum transfer). The onset of the carbon *K*-edge is marked by the horizontal line at 290 eV energy loss.

Contributions from all processes described above exist in the recorded scattering signal and XRS spectra are typically superimposed by the response of the valence electron system and of other core electrons. This is demonstrated in Fig. 2(*a*) ▸ for the case of polycrystalline diamond with 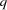 = 15.3 Å^−1^, which is considered often to be large enough for the IA to be valid within reasonable accuracy. The carbon *K* near-edge is visible at an energy loss of ∼282 eV. The valence Compton profile, *i.e.* Compton scattering from the valence electron system, which is undesired background in XRS, is centered around 950 eV. This valence Compton profile constitutes a highly non-trivial background to the core-electron spectrum. The extraction of the XRS core spectrum from the superposition of the core and valence Compton profiles is described in detail in §5.

Fig. 2(*b*) ▸ aims to clarify how the valence-electron spectrum behaves with respect to a core-level excitation. It shows the dispersion of the valence electron response, which is centered at the plasmon energy 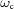 at the region we refer to as ‘low’ momentum transfer (Huotari *et al.*, 2011*a* ▸). It follows a roughly quadratic behavior 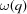 = 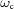 + 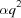 + 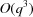 until the ‘medium’ momentum transfer region. At even higher values of *q*, the spectra start following the energy transfer expected for the Compton shift, 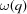 = 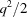. This is the so-called ‘high’ momentum transfer region.

We also wish to note that an excellent overview of XRS is given in chapters 3 and 6 of Schülke (2007 ▸). Comprehensive reviews of inelastic X-ray scattering can be found by, for example, Hämäläinen & Manninen (2001 ▸), Sinha (2001 ▸) and Sternemann *et al.* (2008*a* ▸).

## 3. Planning XRS experiments   

The small scattering cross section and the momentum transfer sensitivity of XRS require careful planning of XRS experiments. First of all, the subtraction of the valence contribution from the total spectrum is a delicate issue and can complicate data analysis considerably, especially if the desired core edge lies at the peak of the Compton profile. Dealing with this is described in detail in §5 of this article. The easiest way to avoid this difficulty, however, is to choose momentum transfers that guarantee that the binding energies of the core edges of interest are not near the position of the Compton peak maximum.

To this end, it is desirable to construct predictions of experimental spectra, *i.e.* of the Compton profile with respect to the expected core excitations. This can easily be achieved based on tabulated Hartree–Fock (HF) atomic Compton profiles (Biggs *et al.*, 1975 ▸) that can be retrieved from http://ftp.esrf.eu/pub/scisoft/xop2.3/DabaxFiles/ComptonProfiles.dat. These Compton profiles are calculated directly from the electron ground-state wavefunctions and are exact in the limit of the IA. We use the tabulated HF Compton profiles not only for the high *q* and high-energy transfer regimes but for all *q* and ω values accessible in typical XRS experiments. The justification for this approximation is discussed below.

The tabulated and corrected Compton profiles for each electronic shell are brought to energy-loss scale *via* equation (10), cut at the respective electron binding energies, converted to 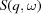 *via* equation (9), and are then normalized to satisfy the *f*-sum rule (Johnson, 1974 ▸),
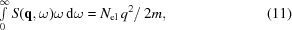
where *m* is the electron mass. The resulting spectra for each shell are then summed up to the total, core and valence contributions to the DDSCS.

To mimic real experimental sample conditions, sample self-absorption is accommodated by the factors 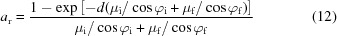
(in reflection geometry) and 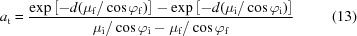
in transmission geometry (Sternemann *et al.*, 2008*b* ▸). Here, *d* is the sample thickness, 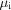 and 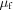 are the energy-dependent absorption coefficients and 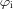 and 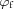 the angles between the sample’s surface normal and the incoming and scattered X-rays, respectively. The values for 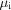 and 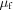 can be derived from tabulated values (McMaster *et al.*, 1969 ▸).

**Figure 3:**
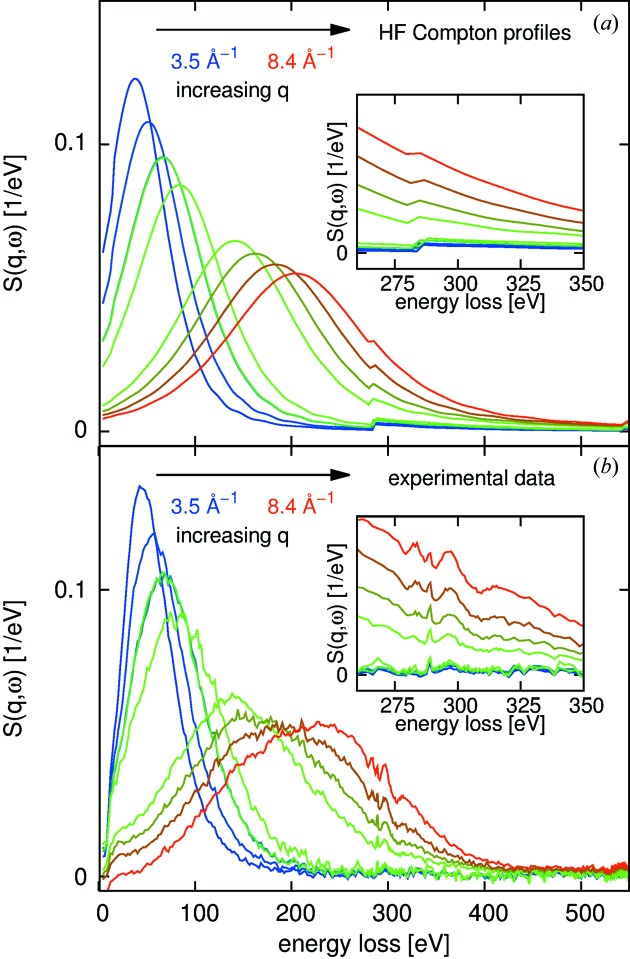
(*a*) Predicted and (*b*) measured spectra for a sample of gaseous acetic acid. The edge jumps and the position of the electron-hole and valence Compton maxima are well reproduced by the simple atomic HF Compton profiles. The two insets show a zoom into the carbon *K* near-edge region for both the predicted spectra (top) and the measured ones (bottom).

The use of the HF Compton profiles also in the low momentum transfer limit is only justified if it serves the purpose of predicting approximate energy positions of the valence electrons’ response and the relative edge jumps. To test this approach, we use data taken from gas-phase acetic acid (CH_3_CO_2_H) measured on ID20 at the ESRF as an example. A comparison of the thus estimated spectra with experimental data is shown in Fig. 3 ▸. Here, indeed we can observe a good correspondence of the predicted and measured signals. The aim of this approach is to estimate the overall spectral shape and predict the signal-to-background ratio. The strength of this method is that the HF Compton profiles are readily available in tabulated form and estimates can be calculated in real time. The HF spectra are naturally not expected to reproduce the measured near-edge structures, which are governed by the geometrical and electronic structure of the absorber’s local environment.

If this was the goal, a more accurate approach *via* calculation of spectra from atomic configurations, *e.g.* using the *FEFF* (Soininen *et al.*, 2005 ▸; Mattern *et al.*, 2012 ▸) or *ERKALE* (Lehtola, 2012 ▸; Lehtola *et al.*, 2012 ▸) code, would be desirable. That, however, requires a detailed knowledge of the atomic-level structure of the sample beforehand. This information often is not at hand, since obtaining it is usually the purpose of the experiment in the first place.

Recently, Mattern & Seidler (2013 ▸) have systematically investigated how different approaches to simulating core edge onsets in non-resonant IXS experiments are modeled. According to their findings, simple truncation of HF atomic Compton profiles is indeed a reasonable approximation at higher momentum transfers. Further development to account more accurately also for the low momentum transfer regime is still necessary.

An important question in planning XRS experiments is to which level of dilution a sample can be studied with a meaningful signal-to-noise ratio. Compared with X-ray absorption spectroscopy, where concentrations down to the parts-per-billion regime (as, for example, in Wilke *et al.*, 2012 ▸) can be measured using fluorescence detection, XRS is less sensitive to low concentrations and an estimate prior to an experiment is often crucial. For this estimation, the conversion of 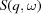 to intensity is necessary and the finite analyzer reflectivity and detector efficiency have to be taken into account. Following Moretti Sala *et al.* (2013 ▸) we can write the detected photon flux *I* as 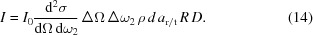
Here, 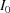 is the incident photon flux, 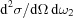 is the DDSCS from equation (1), 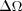 is the solid angle of detection, 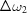 is the energy resolution, ρ is the number density of scatterers in the interaction volume, *d* is the sample thickness, 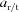 is the absorption factor of equations (12) and (13), and *R* and *D* are the finite reflectivity of the analyzer crystals and the detector efficiency, respectively.

**Figure 4:**
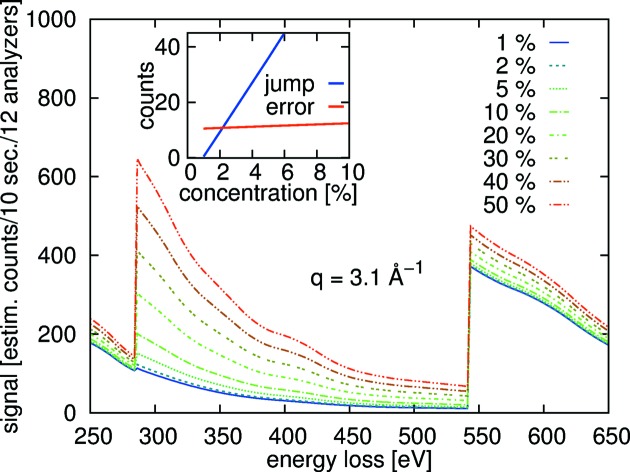
Estimated signal *I* from different concentrations of mixtures of water and acetic acid for 12 analyzer crystals of 10 cm diameter (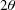 = 35° in transmission geometry, 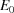 = 9.68 keV, sample thickness 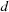 = 1 mm).

As a demonstration, Fig. 4 ▸ shows a count rate prediction for a series of different concentrations of acetic acid in water. For this estimate, we used a scattering angle of 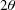 = 35° in transmission geometry, and set the analyzer energy to 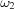 = 9.68 keV [*i.e.* the Si(660) reflection]. The sample thickness was 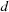 = 0.1 cm, and the densities were set to 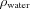 = 1.00 g cm^−3^ and 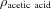 = 1.05 g cm^−3^. The incident flux was assumed to be 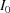 = 10^13^ photons s^−1^, 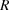 = 0.2 and 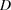 = 0.85. The efficiency of the detector follows from 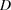 = 1 − 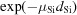, with the absorption coefficient 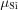 of the detector active material and its thickness 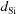 = 500 µm [as in the Maxipix detector (Ponchut *et al.*, 2011 ▸)]. For an estimate of the analyzer reflectivity, we solve the Takagi–Taupin equations (Takagi, 1962 ▸; Taupin, 1964 ▸; Vartanyants *et al.*, 1993 ▸) for a spherically bent silicon Si(660) crystal analyzer. We approximate *R* in (14) by averaging over the full width at half-maximum of the resulting reflectivity curve. The final estimates shown in Fig. 4 ▸ follow by multiplying the resulting *I* by a factor of ten to mimic 10 s counting time per point and a factor of 12 to mimic the sum obtained from 12 individual analyzer crystals. The inset of Fig. 4 ▸ shows the estimated edge jumps in *I* and the statistical accuracy at the carbon *K*-edge as a function of concentration. Taking this example of one scan lasting ∼20 min, meaningful carbon *K*-edge spectra of acetic acid in water can be recorded from solutions of *ca* 2 mol.%. The detection limit will scale as a square root of the time spent in the measurement, and it can be estimated to reach ∼0.4 mol.% in one shift of 8 h.

Overall, estimating the obtainable statistical accuracy and time required for an XRS experiment has not been easy up to now owing to the complex dependence of the XRS signal and the background on the elemental composition of the specimen, energy transfer and momentum transfer. The presented methodology makes this task feasible even in complex samples.

## 4. Performing XRS experiments   

For setups with multiple analyzer crystals, it is desirable to evaluate the spectra from each analyzer crystal separately as they may vary in momentum transfer, energy resolution and/or background and efficiency. This goal can be achieved by using two-dimensional detectors onto which scattering from each analyzer crystal can be focused to a separate spot of the detector area. Here, a region of interest (ROI) has to be chosen for each of the analyzers’ foci, and intensities from pixels within each of these ROIs should be evaluated separately. For a small number of analyzer crystals it is easily possible to choose these ROIs manually. However, as the number of analyzer crystals increases, this task becomes more and more tedious calling for more sophisticated methods.

**Figure 5:**
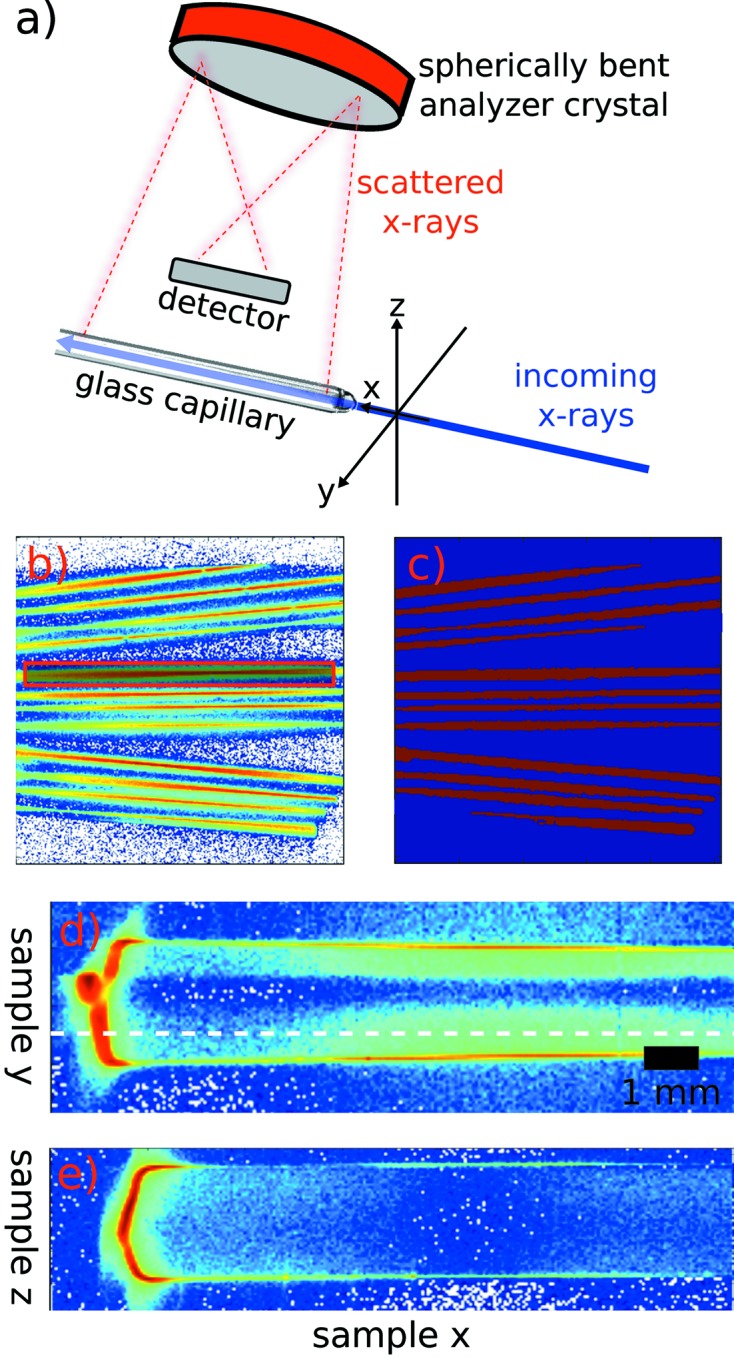
A demonstration of the automatic ROI detection. (*a*) Schematic drawing of the experimental setup used to reconstruct the images in parts (*d*) and (*e*) of this figure. It also shows the setup geometries and laboratory frames we use for the detailed description in the text. (*b*) Original detector image, showing 12 (extended) analyzer foci of a gas sample (intensity is shown on a logarithmic scale). (*c*) Automatically detected ROIs (ROIs in red). (*d*) Two-dimensional reconstruction of a sample position scan along the horizontal direction perpendicular to the X-ray beam (*y*) and (*e*) reconstruction of a sample position scan along the vertical direction (*z*).

The imaging principle and the used coordinate system are shown in Fig. 5(*a*) ▸. In short, we depict a sample that is a high-pressure gas contained inside a glass capillary (diameter 2 mm), aligned parallel to the incident photon beam. These incident X-rays enter through the tip of the capillary and traverse through the entire capillary (length several cm). Twelve analyzer crystals reflect the scattered radiation and focus it on the two-dimensional detector, each one of them onto a different line. Thus the task is to find 12 long lines, containing eventually the measured signals, from the detector images. Fig. 5(*b*) ▸ shows an example of a detector image taken during an elastic line scan using the described setup. The 12 spots, one from each of the 12 analyzer crystals of a single analyzer module at the XRS endstation of ID20 at the ESRF, are elongated across the entire detector width [see Fig. 5(*a*) ▸].

A simple way of finding ROIs automatically is to median-filter (Marion, 1991 ▸) a detector image with all desired focal points and discard pixels which have intensities under a certain threshold. The ROIs automatically detected from the detector image shown in Fig. 5(*b*) ▸ using this scheme are shown as red areas in part (*c*) of the same figure. After identifying the ROIs, the task is to integrate the intensity under each ROI for each image taken during an energy-loss scan, yielding the scattered photon intensity.

The use of two-dimensional detectors also simplifies tremendously the alignment of samples contained in such complicated *in situ* sample environments, including diamond anvil cells for high-pressure experiments. Utilizing the imaging properties of the analyzer crystals (Huotari *et al.*, 2011*b* ▸), one can already use the signal from a single analyzer crystal to reconstruct two-dimensional images from a sample alignment scan. This is illustrated for the XRS experiment on acetic acid (compare Fig. 3 ▸). The schematic scattering geometry used is schematically depicted in Fig. 5(*a*) ▸. The constructed images from alignment scans along the *y*- (Fig. 5*d* ▸) and *z*-directions (Fig. 5*e* ▸) allow visualization of details within the probed sample volume. For the reconstruction of these images, we used the one-dimensional piercing mode as described by Huotari *et al.* (2011*b* ▸). For example, one can clearly see a small droplet of glass in the center of the closed end of the 2 mm capillary and the shadow it produces within the capillary. Ideally, the measurement should be performed above or below this shadow. Fig. 5(*e*) ▸ depicts a stacking of images of a single linear ROI [red shaded box indicated in Fig. 5(*b*) ▸] from a vertical alignment scan (sample translation along the *z*-axis in the laboratory frame) of the same capillary. The scan was performed at a sample *y*-position as indicated in Fig. 5(*d*) ▸.

As demonstrated above, the focusing properties of the analyser crystals give access to imaging (so-called direct tomography) of the sample and its environment. Especially in experiments involving complex sample setups, such as a high-pressure cell, this is an invaluable tool even for visualization and alignment purposes. More importantly, it has been shown (Huotari *et al.*, 2011*b* ▸) that this even yields access to imaging of heterogeneous sample systems using XRS as a contrast mechanism. This will be discussed further in §6.

## 5. Analyzing XRS data   

Once raw data from several energy scans are summed for each analyzer, care has to be taken to extract the desired XRS signal from the collected data. The underlying background is mainly due to the response of the valence electrons and, to a lesser extent, to stray scattering (mainly elastic scattering and fluorescence radiation).

Stray scattering and detector noise can be estimated by measuring scattering at energies below the elastic line, or, in the case of two-dimensional detectors, by defining one extra ROI away from the analyzer foci. The signal from this background estimation should be fitted by a constant or linear function, extrapolated to all relevant energies, and subtracted from the data.

Sternemann *et al.* (2008*b* ▸) and Huotari *et al.* (2012 ▸) have outlined a procedure for the extraction of the valence Compton profile and demonstrated it for the case of polycrystalline Si and SiO_2_ samples and polycrystalline diamond, respectively. Here, we use the same approach as described in the two articles.

Even though the tabulated atomic HF Compton profiles proved good enough to estimate the position of the valence electrons’ response and approximate core edge jumps (see §3), the valence Compton profiles in real systems are far from atomic ones and the tabulated atomic Compton profiles cannot be used to subtract this contribution to extract the desired core edge spectra. However, in the case of medium and high *q*, Sternemann *et al.* (2008*b* ▸) and Huotari *et al.* (2012 ▸) showed that if the valence Compton profile can be measured in a way that it is not obscured by the desired or other core excitations [*cf*. Fig. 2(*b*) ▸ for the example of carbon], this valence profile then reflects the valence electron contribution at other *q* well enough for extraction purposes. When extracted, this valence Compton profile can be subtracted from all other measured data at arbitrary medium to high momentum transfers.

**Figure 6:**
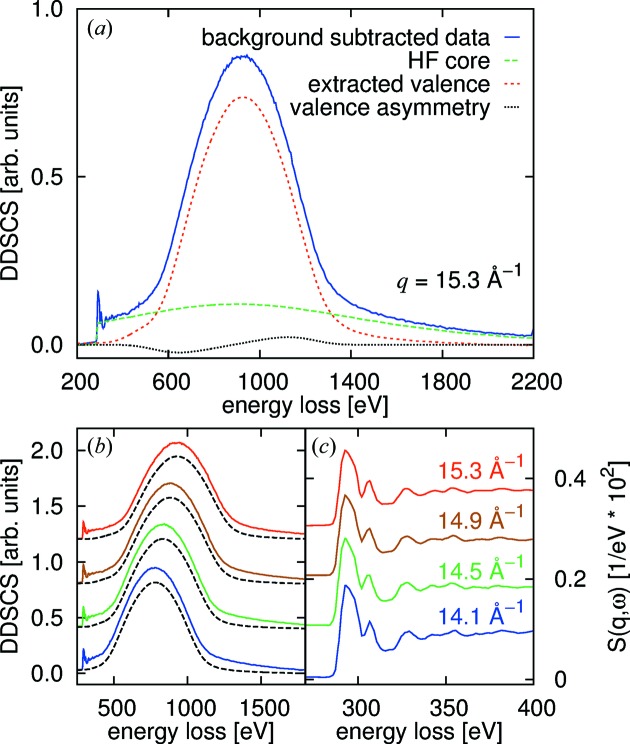
Valence Compton profile extraction scheme demonstrated for experimental data of polycrystalline diamond. (*a*) Recorded experimental data at a momentum transfer of 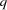 = 15.3 Å^−1^, the corresponding HF core Compton profile, the extracted and asymmetry-corrected valence Compton profiles, and the fitted asymmetry. (*b*) Measured data from different *q*-values, and the valence profile from the highest *q*-value, transferred to the respective momentum transfer (increasing *q* from bottom to top). (*c*) Extracted near-edge spectra from the data of (*b*) [the according momentum transfers shown here are refer also to the spectra shown in part (*b*)].

An example of this extraction scheme for spectra of polycrystalline diamond at high and medium *q*-values is provided in Fig. 6 ▸. The experimental data for this demonstration were taken from Huotari *et al.* (2012 ▸). First, we subtracted a linear background from the experimental data. Sample self-absorption and absorption of the incident X-ray beam between the incident flux monitor and the sample were corrected for. The spectra were brought onto absolute scale using the relativistic cross section (*cf*. Sternemann *et al.*, 2008*b* ▸). Next, we subtracted the HF core Compton profile. If the valence Compton profile is obscured by near-edge oscillations (as is the case here near the carbon *K*-edge at 300 eV), the obscured part can be replaced by a Pearson VII function. The resulting valence Compton profile is usually asymmetric since, even at the relatively high momentum transfer values used to record it, the impulse approximation is not strictly valid (Huotari *et al.*, 2001 ▸). We correct for this asymmetry by fitting a phenomenological asymmetry function 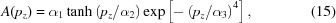
which is shown together with the resulting valence Compton profile in Fig. 6(*a*) ▸. Using the definition of 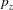 [equation (10)], the extracted valence profile and fitted asymmetry are then transferred to the other high and medium momentum transfers. Fig. 6(*b*) ▸ shows the measured data and predicted valence Compton profiles for four different *q*-values. The valence Compton subtracted near-edge data are shown in Fig. 6(*c*) ▸.

At low momentum transfers, the extracted valence Compton profile is no longer a good estimate of the background since here the spectrum is dominated by particle–hole and/or plasmon excitations and not Compton scattering. Here, it is most straightforward to use parametrized functions, such as a Pearson VII, linear or even a constant function, to mimic the background.

Often, the experimental spectra are superimposed by a number of other absorption-edge onsets so that the described extraction scheme fails and the background can only be estimated from a few tens of eV below the edge onset. In these cases, the edge jump is generally evident from the data, but it is usually difficult to estimate the slope of the spectra tens of eV above the edge onset.

**Figure 7:**
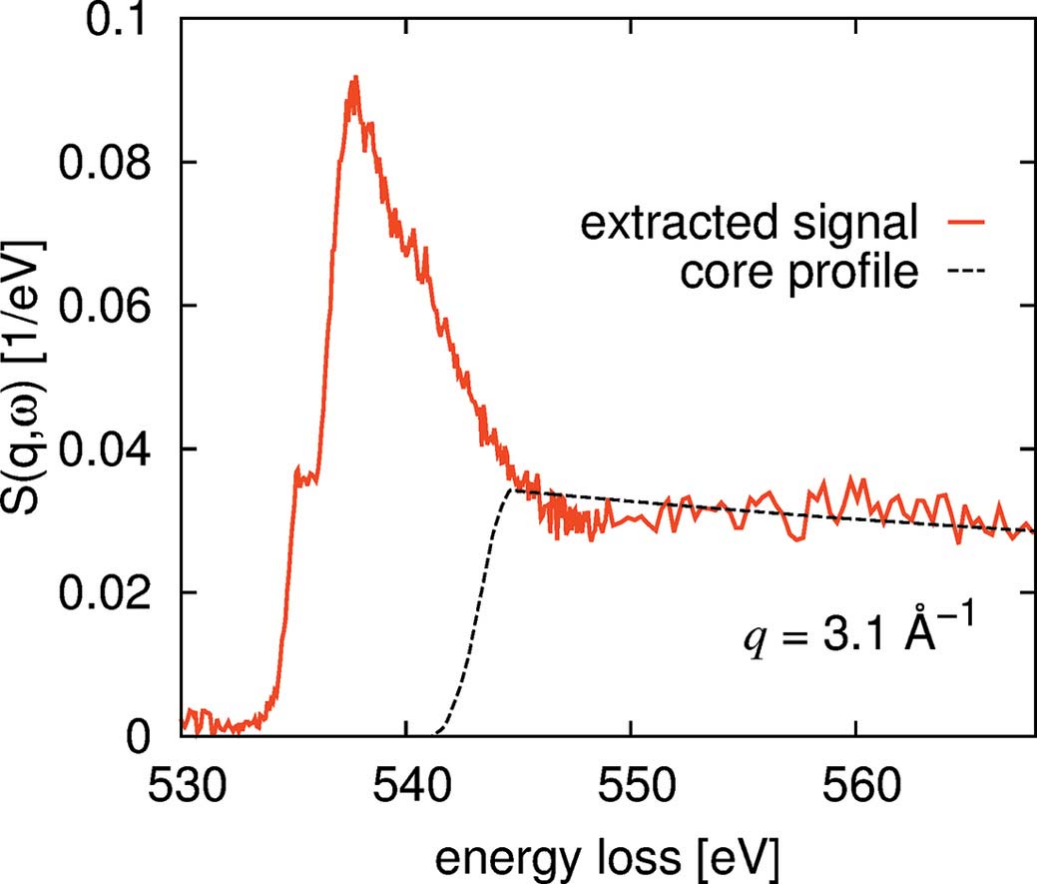
Low-*q* extraction and normalization with HF Compton profiles. The figure shows a spectrum of the oxygen *K*-edge of a 15 *M* LiCl solution.

Also in this case, the parametrized HF Compton profiles prove to be valuable. These profiles are by definition normalized to the *f*-sum rule which provides an absolute scale for the measured data. Thus, we utilize these HF Compton profiles to subtract background functions, such as Pearson VII, linear or constant, under the constrain that the measured data oscillate around the HF core Compton profile further above the edge onset. An example of this procedure is shown in Fig. 7 ▸ for the oxygen *K*-edge from a 15 molar aqueous LiCl solution [data taken from Juurinen *et al.* (2013 ▸)]. Here, a constant background before the edge was assumed. An advantage of this procedure is that even data that are noisy and/or are measured over a limited energy range can be normalized and background-subtracted to yield spectra which are comparable on an absolute scale.

## 6. Imaging with XRS contrast and super-resolution   

As was demonstrated by Huotari *et al.* (2011*b* ▸), the imaging properties of bent crystal analyzers can be used for direct tomography with chemical bond contrast. With this technique, studies of the local structure and chemical bonding of low-*Z* elements become accessible enabling *in situ* studies of chemistry and physics in 3D.

However, the spatial resolution achievable with current setups is modest and limited by the dimensions of the incoming X-ray beam, the size of the analyzer crystal foci, and the finite detector pixel size. The incident beam focus and the quality of the analyzer crystals is determined by the beamline and the spectrometer. The limitations of a finite detector pixel size, however, can be circumvented using the super-resolution approach, which is commonly used in other imaging techniques such as MRI, CT, optical microscopy and digital photography (Park *et al.*, 2003 ▸). In the super-resolution technique, several low-resolution, noisy or blurred images are combined to produce a higher-resolution image (Farsiu *et al.*, 2004 ▸). A prerequisite is the existence of aliasing, *i.e.* the existence of different signals that are indistinguishable in the low-resolution images. This aliasing can be removed if there is a relative sub-pixel misalignment between the low-resolution images. Data from multi-analyzer-crystal setups are predestined for this approach since they inherently provide images of the same object (the sample) seen from different perspectives, *i.e.* each analyzer provides a low-resolution image all of which can then be used in the construction of a super-resolution image.

Two steps are required to construct a super-resolution image from a series of low-resolution images. The first one is the image registration, *i.e.* the estimation of sub-pixel shifts imposed on the images by either sub-pixel size translation of the detector or, as mentioned before, different perspectives from which the low-resolution images were taken. The second step is the (non-linear) interpolation of the set of low-resolution images to construct a high-resolution image.

**Figure 8:**
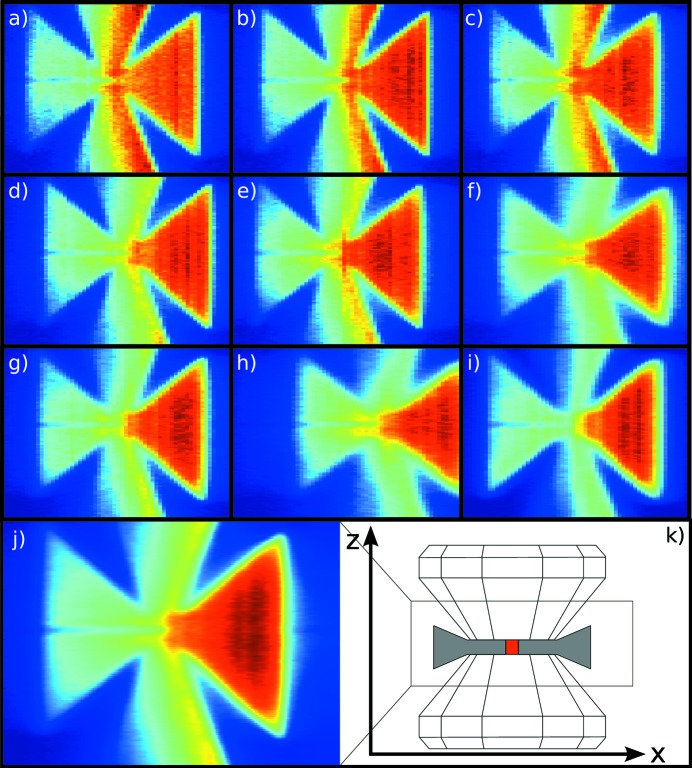
Direct tomography images of a diamond anvil cell. (*a*)–(*i*) Nine low-resolution images (one from each analyzer crystal). (*j*) Super-resolution image constructed from the those nine low-resolution images. The number of pixels along the *x*-direction was increased by a factor of four in the super-resolution image as compared with the low-resolution images. (*k*) Schematic drawing of a diamond anvil cell that shows the scanned area.

For an example of this super-resolution approach, Fig. 8 ▸ shows two-dimensional sections of a diamond anvil cell, such as is used in high-pressure studies (Tse *et al.*, 2011 ▸, 2014 ▸; Sternemann *et al.*, 2013 ▸; Rueff & Shukla, 2010 ▸). The low-resolution images [Figs. 8(*a*)–8(*i*) ▸] were obtained by scanning the height (*z*) of the diamond anvil cell at the maximum of the quasi-elastic line at 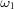 = 9.68 keV using the 9-analyzer crystal spectrometer of ID16 of the ESRF (Verbeni *et al.*, 2009 ▸). Thus, the contrast for these images is given by the static structure factor 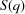. All nine low-resolution images share a common *z*-axis. The *x*-axes were scaled to correct for the different scattering angles of each analyzer crystal. The shifts, on the other hand, were estimated by a least-square minimization of the pixel-by-pixel difference of all low-resolution images with respect to the first one by variation of the shift along the *x*-axis only. The images were then interpolated onto a grid with a fourfold number of pixels in the *x*-direction compared with the low-resolution images. The resulting super-resolution image is shown in Fig. 8(*j*) ▸. Three-dimensional data can then be obtained by stacking resolution-enhanced images [such as Fig. 8(*j*) ▸] taken at different positions in the *y*-direction [perpendicular to both the *x*- and *z*-directions indicated in Fig. 8(*k*) ▸]. Besides the gain in spatial sampling, the alignment of low-resolution images also improves the statistical accuracy for direct tomography. Combining images from several analyzer crystals thus shortens the required exposure time considerably.

As was noted above, multiple-crystal spectrometers designed for XRS are perfectly suited to the use of the super-resolution technique, especially since low-noise photon-counting two-dimensional pixel detectors with spatial resolution below 55 µm are not readily available. This approach lifts the limitation on the detector pixel size to the obtainable image quality of the direct tomography method. It should be noted that the final resolution will be limited by the charge cloud size on the pixel detector (decreasing pixel size below that level has little gain), and the focusing properties of the analyzer crystals. Recent developments on the latter (Verbeni, 2014 ▸) and improved understanding of spherically bent analyser crystal diffraction properties (Honkanen *et al.*, 2014*a* ▸,*b* ▸) are paving the way for an efficient use of XRS-based direct tomography.

## 7. Conclusions and outlook   

In this article we have described how we plan and perform X-ray Raman scattering experiments and analyze data from such experiments. Owing to the momentum transfer dependence of XRS and the fact that the measured cross sections are superimposed by the response of the valence electron system, careful planning of XRS experiments is necessary. We demonstrated how to assess optimal scattering geometries and sample geometries (such as concentrations and thicknesses) by use of tabulated Hartree–Fock Compton profiles. We showed how to utilize two-dimensional detectors for easy sample alignment even if the sample is contained in complicated sample environments and easy analyzer-by-analyzer data evaluation. The use of the tabulated Hartree–Fock Compton profiles also simplifies the extraction of the desired core edge spectra. Using two-dimensional detectors also allows for direct tomography and we outlined how such direct tomography images constructed from different analyzer crystals can be combined to produce a resolution-enhanced image.

To simplify all steps described in this article, we developed an open-source software package, which is intended to simplify XRS data collection and analysis of experimental data from the new multi-analyzer-crystal inelastic scattering beamlines such as ID20 at the ESRF. This open source tool is written in the Python programming language and is distributed as a Python module (http://www.github.com).

Future developments of the code should include a more proper treatment of the low momentum transfer regime. Integrating functionality for the extraction of the partial local projected densities of states (l-DOS) as demonstrated by, for example, Soininen *et al.* (2005 ▸) is planned as a future development. Extending the program for the analysis of other regimes of non-resonant scattering, *e.g.* valence electron excitations, and other inelastic X-ray scattering techniques, such as RIXS, is also desirable.

## Footnotes

1 ID16 was closed for operation in 2012 and the IXS spectroscopy research of electronic excitations continues at the new beamline ID20 as a part of the ESRF Upgrade Programme.
